# Classification of tomato leaf disease using Transductive Long Short-Term Memory with an attention mechanism

**DOI:** 10.3389/fpls.2024.1467811

**Published:** 2025-01-21

**Authors:** Aarthi Chelladurai, D.P. Manoj Kumar, S. S. Askar, Mohamed Abouhawwash

**Affiliations:** ^1^ Department of Electronics and Communication Engineering, Sengunthar Engineering College, Tiruchengode, India; ^2^ Department of Computer Science and Engineering, Kalpataru Institute of Technology, Tiptur, India; ^3^ Department of Statistics and Operations Research, College of Science, King Saud University, Riyadh, Saudi Arabia; ^4^ Department of Animal Science, Michigan State University, East Lansing, MI, United States; ^5^ Department of Mathematics, Faculty of Science, Mansoura University, Mansoura, Egypt

**Keywords:** attention mechanism, data augmentation, segmentation, tomato leaf disease, Transductive Long Short-Term Memory

## Abstract

Tomatoes are considered one of the most valuable vegetables around the world due to their usage and minimal harvesting period. However, effective harvesting still remains a major issue because tomatoes are easily susceptible to weather conditions and other types of attacks. Thus, numerous research studies have been introduced based on deep learning models for the efficient classification of tomato leaf disease. However, the usage of a single architecture does not provide the best results due to the limited computational ability and classification complexity. Thus, this research used Transductive Long Short-Term Memory (T-LSTM) with an attention mechanism. The attention mechanism introduced in T-LSTM has the ability to focus on various parts of the image sequence. Transductive learning exploits the specific characteristics of the training instances to make accurate predictions. This can involve leveraging the relationships and patterns observed within the dataset. The T-LSTM is based on the transductive learning approach and the scaled dot product attention evaluates the weights of each step based on the hidden state and image patches which helps in effective classification. The data was gathered from the PlantVillage dataset and the pre-processing was conducted based on image resizing, color enhancement, and data augmentation. These outputs were then processed in the segmentation stage where the U-Net architecture was applied. After segmentation, VGG-16 architecture was used for feature extraction and the classification was done through the proposed T-LSTM with an attention mechanism. The experimental outcome shows that the proposed classifier achieved an accuracy of 99.98% which is comparably better than existing convolutional neural network models with transfer learning and IBSA-NET.

## 1 Introduction

In most Asian and African nations, agriculture is one of the primary sources of revenue (Raja Kumar et al., 2023). Plant disease identification and classification are essential for precise agriculture since they increase farmers’ production and raise their level of living. Early plant disease identification and classification can likely reduce farmers’ and the country’s financial losses (Ashwinkumar et al., 2022; Pandian et al., 2022). Since leaves aid in photosynthesis and supply vital nutrients and minerals for plant growth, it is necessary to see leaf diseases as a crucial phase (Espejo-Garcia et al., 2021; Reddy et al., 2023). Plant disease is a major issue that affects production quality and causes economic loss for individuals and society (Sreedevi and Manike, 2023). The diseases are due to bacteria, pathogens, fungi, and virus-like organisms and are a major variable that affects the lifespan of plants (Harakannanavar et al., 2022; Fukada et al., 2023; Singh et al., 2023). Therefore, the use of an automated system facilitates the efficient detection and classification of plant diseases with the application of deep learning and machine learning (ML) models (Mahato et al., 2022; Alzahrani and Alsaade, 2023). The rapid development in the field of ML has presented efficient results for plant disease identification.

The accessibility of cost-effective devices has allowed scholars to take images in real-time and provide superior results through ML. Some of the ML models, which include decision trees (DT) (Kiran and Chandrappa, 2023), support vector machines (SVM) (Sagar and Singh, 2023), and K-nearest neighbors (KNN) (Chong et al., 2023), have been assessed by scholars for classification. These models have a robust design and operate fine with less training data. However, they are incapable of dealing with interferences such as intensity disparities, color variations, and illumination modifications (Borugadda et al., 2023; Tabbakh and Barpanda, 2023). Furthermore, these traditional models have trade-offs between classification and detection (Sanida et al., 2023). The development of deep learning (DL) models has helped scholars overcome the drawbacks of traditional ML models. Numerous DL models such as convolutional neural network (CNN), recurrent neural networks (RNNs) (Chen et al., 2021), and Long Short-Term Memory (LSTM) (Lin and Leong, 2023) models have been found to be dependable in identifying plant diseases (Zhou et al., 2023) and are used in comparative evaluations. Due to these features, DL models have been found to be appropriate in the field of agriculture and plant disease classification (Zhong et al., 2023). The existing techniques for tomato leaf classification face substantial issues such as overfitting, low scalability, and diminished accuracy, especially when working with simulated data and larger databases. These restrictions arise from the existence of noise and insufficient feature extraction methods that struggle to differentiate appropriate features and accordingly, the performance is compromised. Crop disease detection is considered a vital factor of modern agriculture that enables farmers to recognize the diseases that affect crop yields and quality. Therefore, this research aims to address this problem by introducing a scalable and accurate crop disease detection technique by means of DL models (Shahi et al., 2023). This research introduced an effective classification approach called Transductive LSTM (T-LSTM) with an attention mechanism to classify diseased tomato leaves, enhancing the scalability and minimizing the overfitting.

The major contribution of this study is as follows:

1. The pre-processing based on color enhancement, image resizing, and data augmentation was performed and the data was then fed into the segmentation phase that used U-Net architecture.
2. The extraction of features from the segmented output used VGG-16 and finally, the classification of diseased leaves and healthy leaves was conducted using T-LSTM with an attention mechanism.
3. The transductive LSTM with an attention mechanism is introduced to classify tomato leaf disease. The T-LSTM is based on the transductive learning approach and an attention mechanism evaluates the weights of each step based on the hidden state and image patches.

This research work is organized as follows. Section 2 outlines the related works on the classification of leaf disease. The proposed methodology (materials and method) of this study is offered in Section 3. In Section 4, the experimental outcome attained while estimating the efficacy of the suggested framework is provided. Finally, the conclusion in Section 5.

## 2 Related works

Here, studies that focused on the detection of tomato plant leaf disease with their respective limitations are outlined in detail.

Abbas et al. (2021) describe a DL-based technique for detecting tomato leaf disease which employs a conditional generative adversarial network (C-GAN) to produce synthetic photos of tomatoes. Furthermore, this research used the DenseNet121 model to train, and the pre-trained model was fine-tuned on actual and synthetic images. The C-GAN-based augmentation approach improves generalizability and prevents overfitting issues. However, data replication occurs in DenseNet 121 when feature maps are spliced with previous layers. Saeed et al. (2023) developed a smart tomato leaf disease detection method based on transfer learning techniques such as a CNN. The method’s first layer was removed and it was replaced by softmax layers. The suggested method was a better classification approach as the dropout rate was lowered, but random connections of feature maps caused overfitting issues in the CNN.

Ahmad et al. (2021) demonstrated an approach for categorizing leaf disease using a CNN based on the symptoms of leaf disease. Initially, the dataset was evaluated based on class imbalances, and the stepwise transfer learning approach was used to reduce CNN convergence time. The proposed approach was tested using the PlantVillage and pepper disease databases and provided accurate solutions. However, the proposed approach ran into issues with long running times and high computational costs. Wu et al. (2020) used GAN-based data augmentation to improve leaf disease accuracy. Deep convolutional GAN (DCGAN) and GoogleNet has been used to produce augmented images and predict disease. However, there was an inefficiency due to the noise-to-image GANs, which displayed healthy leaves as diseased leaves.

Huang et al. (2023) introduced a fully convolutional-switchable normalization dual path network (FC-SNDPN) to detect the tomato leaf disease. This research utilized a fully convolutional network (FCN) which enhanced the segmentation capability. After this, an improved DPN was utilized for feature extraction and SNDPN was the combination which connected the Dense Net and ResNet layers. The SN layer optimized the parameters of the DPN by switching the normalized layer and helping to enhance the versatility. However, the suggested framework did not suit large datasets due to its constrained architecture. Chen et al. (2022) introduced the AlexNet CNN to detect and classify leaf disease. The CNN algorithm was utilized in the pre-processing stage and classification was performed based on a modified AlexNet to decide the accuracy. The combination of a CNN and AlexNet was fed as an algorithm in a mobile-based platform due to the limited memory capacity. This approach helps users detect the type of disease and manage it at earlier stages. However, the framework is invalid for new mobile devices which considered as the drawback of this approach.

Kaur et al. (2022) introduced a modified Mask Region CNN (Mask R-CNN) for automated segmentation of leaf disease. The suggested framework has a RCNN which helps to conserve the memory. The data pre-processing was performed using light subtraction, reduction of noise, and normalization. The magnitudes of anchor in RPN network helped to enhance the detection accuracy and enhanced the overall performance. However, the effective extraction of features based on color and texture was not considered. Bhujel et al. (2022) introduced a lightweight attention-based CNN for the classification of tomato leaf disease. The suggested approach utilized an attention module which was utilized to minimize the complexity of the CNN during classification. The key features were determined based on the location and the critical features were finalized by the position. The suggested framework was a lightweight module that increases CNN presentation and provided better classification results.

J. Arun Pandian and K. Kanchanadev (Arun Pandian and Kanchanadevi, 2022) introduced a dense convolutional neural network with five dense blocks that was referred to as 5DB-DenseConvNet to detect plant leaf disease. The architecture of the 5DB-DenseConvNet comprised five dense blocks and four transition layers. The dataset magnitude was improvised with the help of different augmentation approaches and a GAN. However, the DenseNet architecture faced issues related to data replication that affected categorization efficiency of the model. Attallah (2023) introduced a leaf disease classification model using a compact CNN along with transfer learning and feature selection. The suggested approach utilized three compact structures of a CNN which included deep layers and minimal parameters to reduce the running time and complexity. However, misclassification occurred while classifying images with complex backgrounds. Zhang et al. (2023) introduced IBSA-Net for disease identification on the basis of transfer learning with small sampled data. IBSA-Net was a combined inverted bottleneck network and shuffle attention model which incorporated a hard swish activation and a IBMax function. The suggested approach extracts the multi-level features and located the disease region with fine granularities. However, misjudgment and inappropriate detection were identified due to growth defects in the tomato leaves.

From the overall findings of the existing research studies, we found that problems occur due to the size of datasets and data duplication when classifying tomato leaves. Furthermore, a issue related to overfitting occurs due to the feature maps and existence of noise that affects the overall efficiency. These challenges highlight the requirement for innovative solutions. Thus, this research introduced an effective classification approach using transductive LSTM with an attention mechanism which is clearly described in the following section.

## 3 Materials and methods

**Figure 1:**
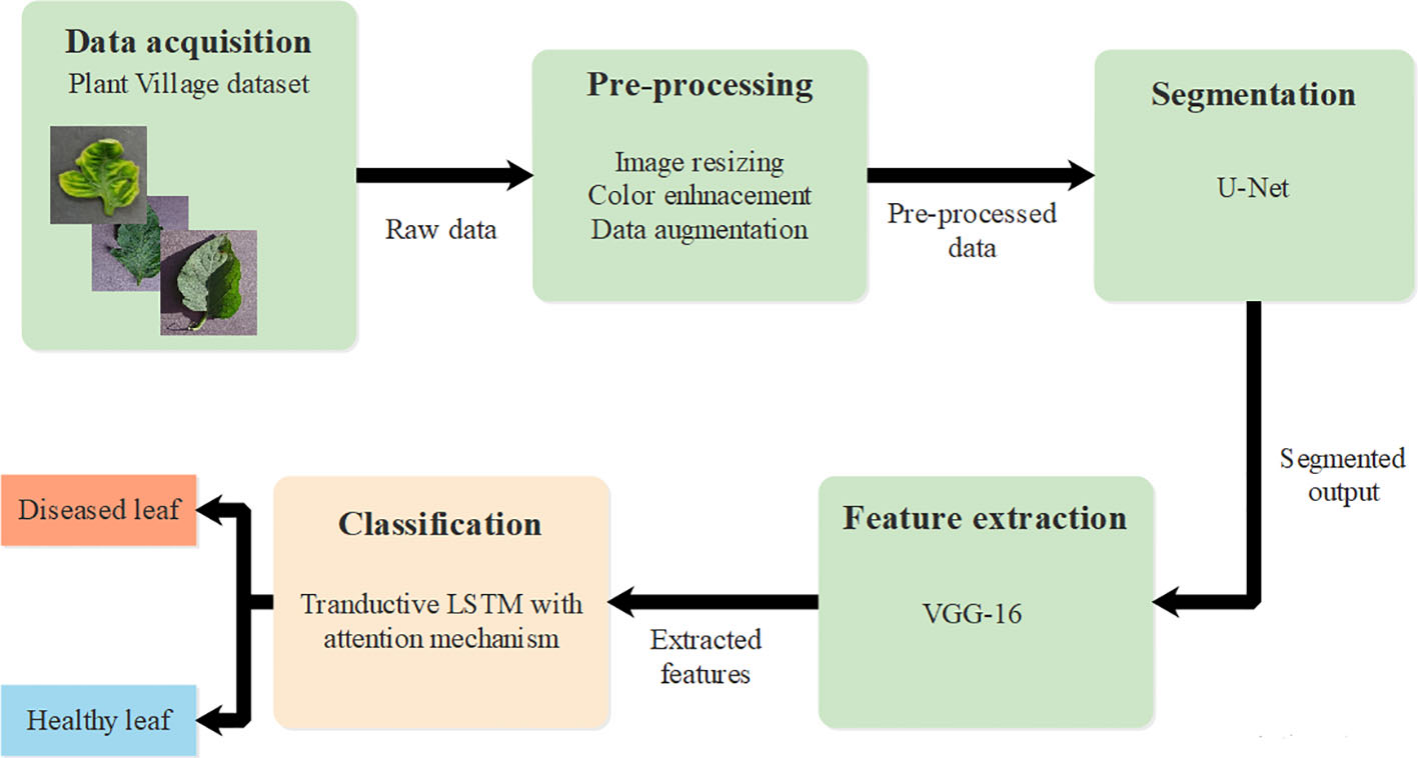
Overall process of tomato leaf disease classification.

This research introduced an effective deep learning classification technique using T-LSTM with an attention mechanism. The proposed approach provides effective classification of tomato leaf disease using T-LSTM which incorporates transductive learning. The T-LSTM has a uniform impact on parameters of the model such as weight and bias. Initially, raw data was obtained from PlantVillage (Dataset) and pre-processing was performed using image resizing, color enhancement, and data augmentation. Segmentation was then performed using U-Net architecture and feature extraction was performed using VGG-16. Finally, classification was accomplished through T-LSTM with an attention mechanism which classified leaves as healthy and diseased leaves. The complete process of tomato leaf disease classification is provided in **Figure 1**.

### 3.1 Data acquisition

The process of data acquisition is the first stage, wherein this research exploits a publicly available dataset known as the PlantVillage dataset (Dataset) which comprises 16,012 leaf images with 10 classesOf these, nine classes are disease-affected tomato leaves and the remaining one class is the healthy class. For effective evaluation, the images were resized to 224×224 . Furthermore, the dataset was divided into training, testing and validation sets in the ratio of 60:30:10. The distribution of the classes present in the dataset is tabulated in **Table 1**.

**Table 1:** Division of classes present in Tomato plant village dataset.

Classes	Number of samples
Early blight	1,000
Bacterial spot	2,127
Septoria leaf spot	1,771
Late blight	1,909
Yellow leaf curl virus	5,357
Target spot	1,404
Tomato mosaic virus	373
Leaf mold	952
Two-spotted spider mites	1,676
Healthy leaves	1,591

**Figure 2:**
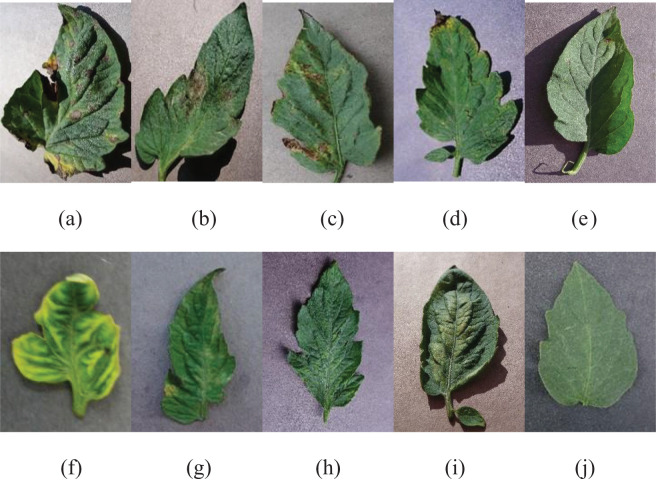
Sample images of tomato leaf disease dataset: **(A)** Early blight; **(B)** late blight; **(C)** bacterial spot; **(D)** Septoria leaf spot; **(E)** target spot; **(F)** yellow leaf curl virus; **(G)** leaf mold; **(H)** tomato mosaic virus; **(I)** two-spotted spider mites; **(J)** healthy leaf.

The data samples gathered from the tomato PlantVillage dataset are presented in **Figure 2**.

### 3.2 Pre-processing

The raw images obtained from the stated database were pre-processed using image resizing, color enhancement, and data augmentation. These stages are briefly discussed below.

#### 3.1.1 Image resizing

Image resizing (Rahman et al., 2023) is a pre-processing technique where the size of the image depends on the input layer size. In this research, an input image of 224×224 was resized to 128×128 . The image resizing standardized the sizes of the images and reduced the computational complexity during classification.

#### 3.1.2 Color enhancement

Color enhancement was used to enhance the visual quality by adjusting the color of the image. In this research, color enhancement was performed using contrast-limited histogram equalization (CLAHE) (Pavan et al., 2023) which generates a realistic form of the image by enhancing the color and brightness of the image.

#### 3.1.3 Data augmentation

Data augmentation (Maliki and Prayoga, 2023) was executed to enhance the size of the training dataset by introducing different transformations such as flipping, rotating, and zooming. The commands random flip, random zoom, and random rotation were utilized to flip the image by reversing the pixel columns, zoom-in, zoom-out, and rotate the image, respectively. Data augmentation helps to maintain the data imbalance during classification.

### 3.3 Segmentation

**Figure 3:**
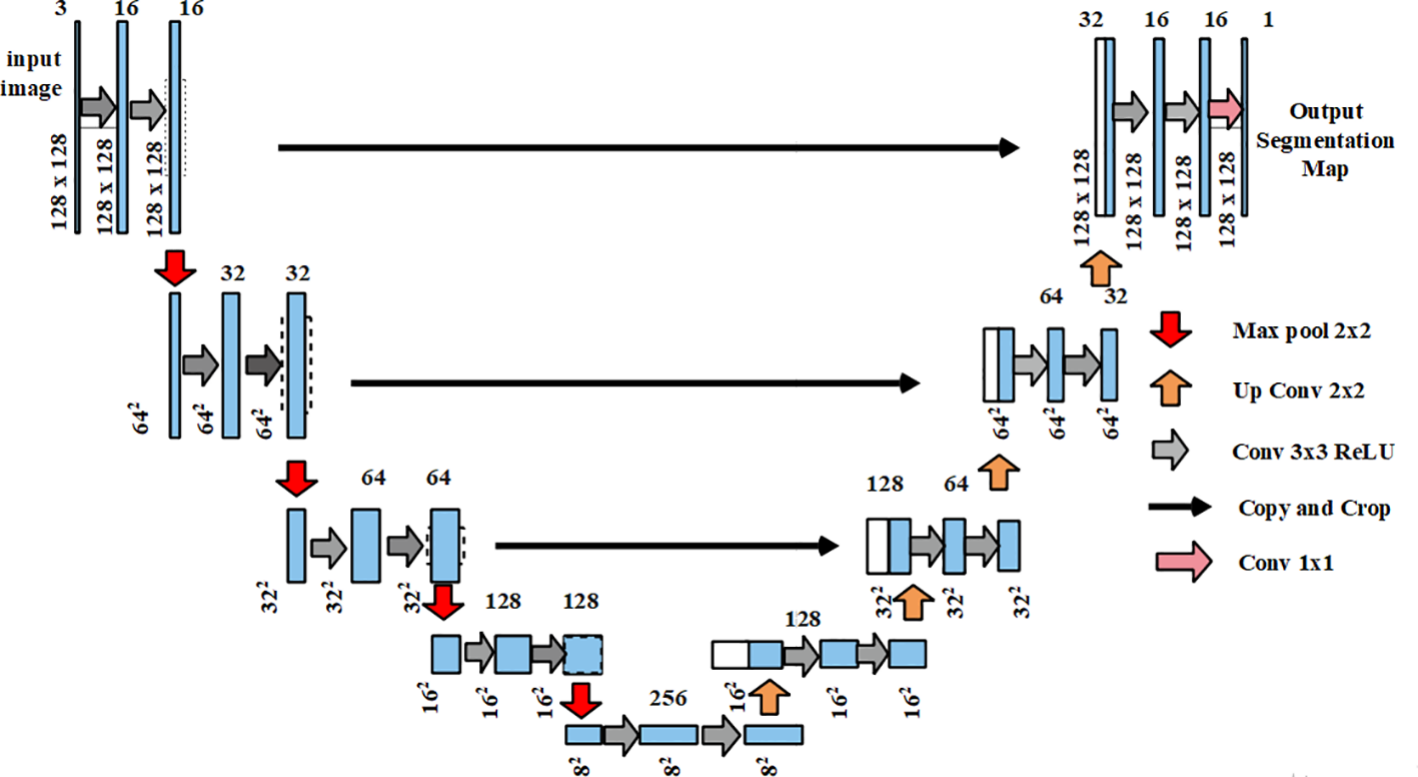
Architecture of U-Net.

The output from the pre-processing stage was provided as the input for segmentation which used U-Net (Yin et al., 2022). The U-Net architecture comprised an expansive path and a contracting path on the right and left sides respectively. Moreover, U-Net comprised two unpadded convolutions of 3×3 continued by a Rectified Linear Unit (ReLU) and a 2×2 maxpooling layer. Every stage in the expansive path comprised upsampling which was followed by a 2×2 convolution that equals the count of the feature channels. In the final layer, a 1×1 convolutional layer was exploited to map the component feature vector of the chosen classes. The U-Net predicts the segmentation masks by adjusting weights based on the predicted output and ground truth values. For the evaluation, the suggested U-Net was analyzed alongside other models such as fully convolutional network (FCN), semantic segmentation network (SegNet), Mask R-CNN, RefineNet, and efficient neural network (ENet) which are described clearly in the result section. **Figure 3** illustrates the architecture of U-Net.

### 3.4 Feature extraction using VGG-16

**Figure 4:**
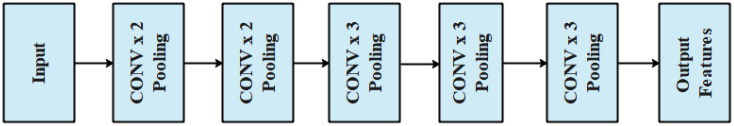
Architecture of VGG-16.

The segmented output was utilized as the input for feature extraction which used VGG architecture. In this research, VGG-16 (Zhu et al., 2022) architecture was used to extract the features that help in the process of classification. VGG-16 is a type of CNN that is capable of extracting deep features. It comprised thirteen convolutional layers with the filter size of 3×3 and the pooling layer size was 2×2 . In general, the structural design helped to adjust the pixel values and helped extract features from the segmented image. The architecture of VGG-16 is presented in **Figure 4**.

The VGG-16 architecture allows us to alter the pixel values of the segmented leaf images and helps in effective feature extraction with its deep convolutional layers. The features obtained from the architecture of VGG-16 were evaluated alongside other feature extraction models such as GoogleNet, AlexNet, ResNet-50, and Inception Net which were then classified by the proposed T-LSTM.

### 3.5 Classification of healthy leaves and diseased leaves using T-LSTM with an attention layer

**Figure 5:**
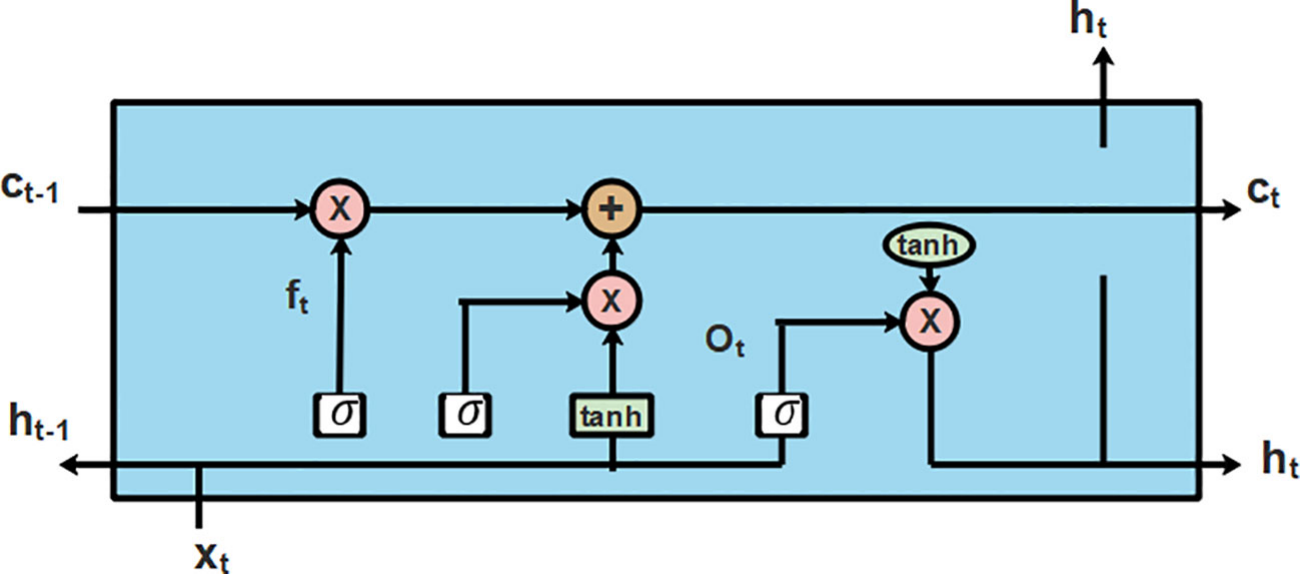
Architecture diagram of LSTM.

An LSTM (Gill and Khehra, 2022) is a particular type of recurrent neural network (RNN) that captures the interclass similarities between extended distances. The architecture of LSTM was selected due to the integrated hidden input and output layers. These layers in the architecture of LSTM have the capability to learn about the features for an ideal prediction by identifying the functional connection from the input data. The structural plan of LSTM is presented in **Figure 5**.

**Figure 6:**
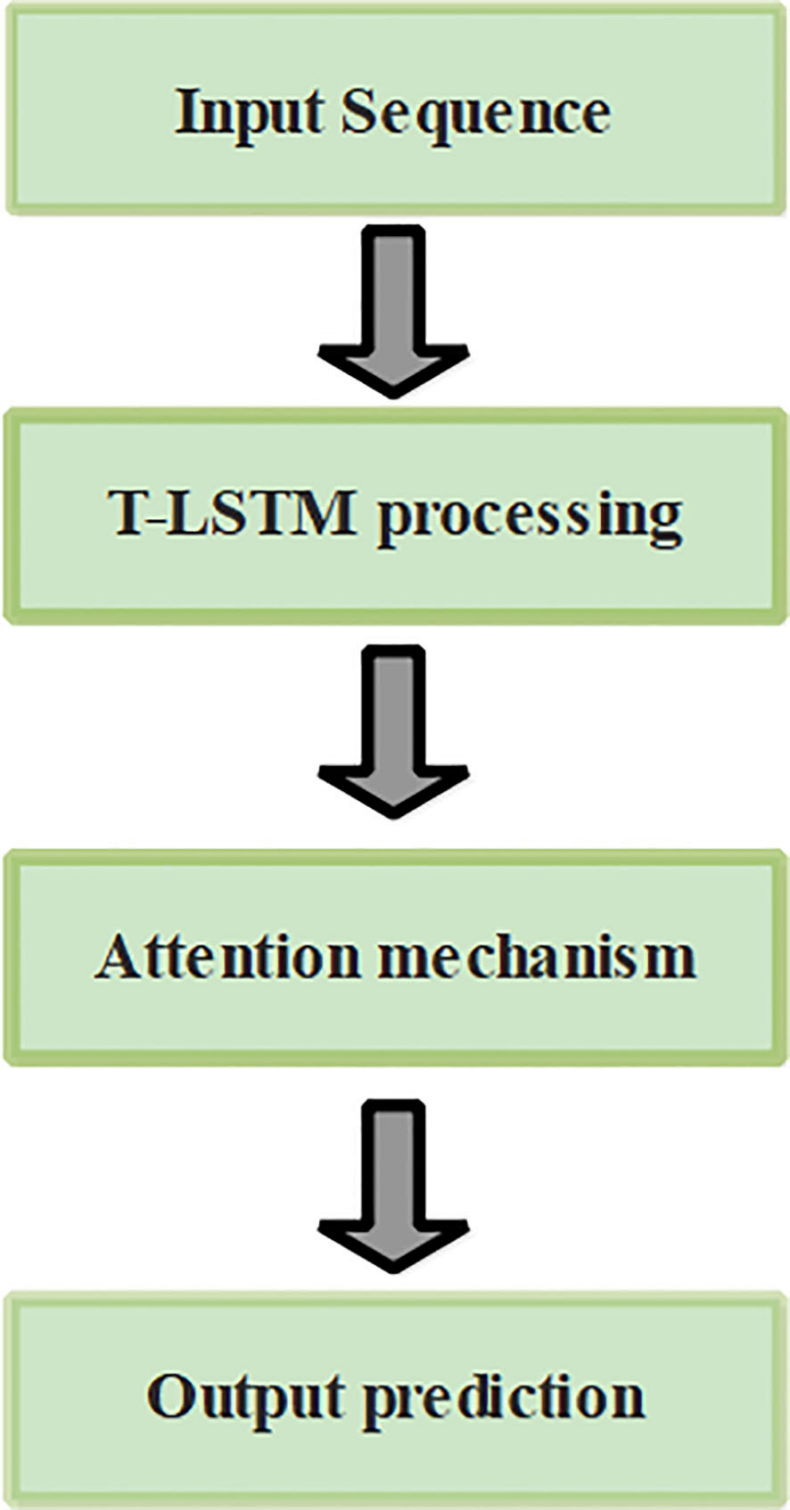
Process of output prediction using T-LSTM with an attention mechanism.

To overcome the aforementioned issues in LSTMs, this research utilized an extended version of LSTM known as T-LSTM. The process leading to output prediction using T-LSTM with an attention mechanism is presented in **Figure 6**.

The training data influenced the recommended features of T-LSTM which were based on the length of the test data point $X^{n}$ referred to as T . The definitive aim of the training phase was to improve the efficacy closer to the test point and enhance the efficiency of the model. Considering a hidden state $K^{\left(n\right)}$ and the state space of T-LSTM model is presented in Equation 1 as follows (Peivandizadeh et al., 2024):


(1) $$\{\begin{array}{c} C_{t,\eta }=f\left(C_{t-1,\eta ,}\;h_{t-1,\;\eta ,}x_{t};\;\omega_{lstm,\eta ,}b_{lstm,\;\eta }\right)\\ h_{t,\eta }=\;g\left(h_{t-1,\eta ,}\;C_{t-1,\;\eta ,}x_{t};\;\omega_{lstm,\eta ,}b_{lstm,\;\eta }\right) \end{array}$$

**Figure 7:**
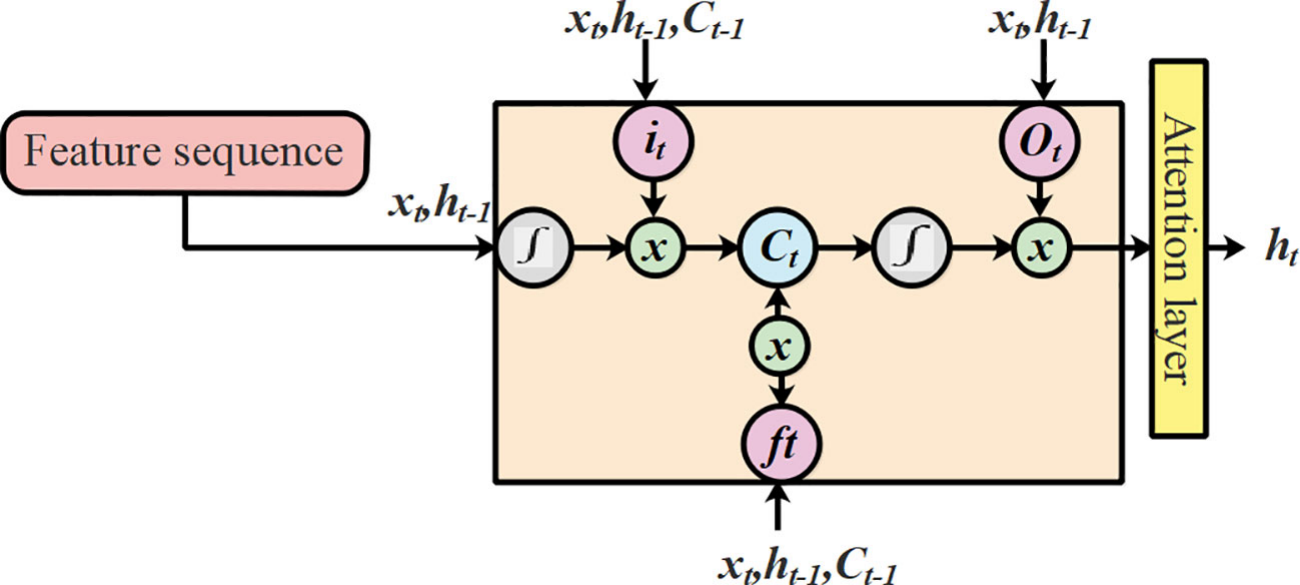
Architecture of T-LSTM with an attention mechanism.

Where *f* (.) and *g* (.) refers to the mapping function of the cell state and the hidden state, respectively. The weighted parameters and the biased parameters are represented as $\omega_{lstm}$ and $b_{lstm}$ . The script value of the sequence $K^{\left(n\right)}$ is represented as  η . The hidden layer is represented as $h_{t}$ , the new input is represented as $x_{t}$ , the previous output is represented as $h_{t-1},$ the cell state is represented as $C_{t}$ , and the cell state at the previous stage is represented as $C_{t-1}$ . The T-LSTM varies from the previous one which was dependent on the feature space of the test points. The structural illustration of T-LSTM is shown in **Figure 7**.

The script value η describes the linear models which were based on data point $X^{n}$ . During the training, the test point function depends on the significance of the data point that was closest to the feature vectors. The prediction performed using the dense layer was based on Equation 2, represented below:


(2) $${\hat{y}}_{\eta }^{\left(t\right)}=\omega_{dense,\eta }^{T}h_{t+T-1,\eta }+\;b_{dense,\eta ,}\;t=1,\ldots ,N$$

Where the weighted and biased term of the dense layer is represented as $\omega_{dense,\eta }^{T}$ and $b_{dense,\eta }$ respectively. The new hidden point is determined by considering the resemblance factor $r_{t,\eta }$ among T which specifies every constraint $\omega_{lstm,\eta ,}\omega_{dense,\eta }$ and $b_{lstm,\eta ,}b_{dense,\eta }$ . The objective function is represented in Equation 3 as follows:


(3) $$({\hat{\omega }}_{lstm,\eta ,}{\hat{\omega }}_{dense,\eta \;}{\hat{b}}_{lstm,\eta ,}{\hat{b}}_{dense,\eta })=\left({\hat{\omega }}_{\eta ,}{\hat{b}}_{\eta }\right)=\arg\text{min}{\;}_{\omega_{\eta ,b_{\eta ,}}}J_{\eta }$$

Where $J_{\eta }=\frac{1}{N}\sum_{t=1}^{N}S_{t,\eta }\;{\left({\hat{y}}_{\eta }^{\left(t\right)}-y^{\left(t\right)}\right)}^{2}+'{ϒ}_{\eta }\omega_{\eta }^{T}\;\omega_{\eta }$ and $S_{t,n}\in R$ , and the tuning parameter is represented as $'{ϒ}_{\eta }$ . The tuning parameter based on the transductive method was exhibited in LSTM which was incapable of employing the training data from the data samples. In the feature space, the distance between the consecutive data points was minimized and the samples from the training phase were obtained prior to the test point. The parameters $\omega_{lstm,\eta ,}\omega_{dense,\eta }$ and $b_{lstm,\eta ,}b_{dense,\eta }$ are dependent on $X^{n}$ so the unseen samples were reformed which shows that the constraint of ${\hat{\omega }}_{\eta ,}{\hat{b}}_{\eta }$ was diverse for each test point. The transductive learning approach trained the LSTM model at each test point which enhanced the model’s precise output prediction ability. The updating portion of the hidden state in $X^{n}$ was be based on Equation 4, as follows:


(4) $$h_{t^{\prime },\eta }=g\left(h_{t^{\prime }-1,\eta ,\;}c_{t^{\prime }-1,\eta ,\;X_{t^{\prime }}^{\left(\eta \right)};}\;{\hat{\omega }}_{lstm,\eta ,\;}\;{\hat{b}}_{lstm,\eta ,\;}\right)$$

Where $t^{\prime }=\eta \ldots ,\eta +T-1$ and the final prediction of output was achieved using Equation 5 as follows:


(5) $${\hat{y}}_{\eta }^{\left(\eta \right)}=\;{\hat{y}}_{dense,\eta }^{T}\;h_{\eta +T-1,\eta }+{\hat{b}}_{dense,\;\eta }$$

Where the input to the hidden state is denoted as $X^{n}$ .

#### 3.5.1 T-LSTM with an attention mechanism

For the given query and the set of key-value pairs, an attention mechanism was presented by query and related keys. The query (Q), key (K) , and value (V) were based on Equation 6. The features are represented as query (Q), the image labels are represented as key (K) , and the value represents input data.


(6) $$A\left(Q,K,V\right)=\sum_{i}\frac{\exp\left(e_{qk_{j}}\right)}{\sum_{j}\exp\left(e_{qk_{j}}\right)}v_{j}$$

Where the alignment model that was used to compute the basic dot product attention is characterized in Equation 7:


(7) $$e_{qk_{j}}=QK^{T}\in R$$

Where $d_{k}=d_{q}$ and the dimensions of the matrix are represented as K and Q correspondingly. The dot product of the attention layer is fast and efficient because it has the ability to be implemented with the help of optimized matrix multiplication. This research utilized a special type of attention mechanism known as scaled dot product attention in a transformer model that is mathematically characterized in Equation 8:


(8) $$A\left(Q,K,V\right)=softmax\left(\frac{QK^{T}}{\sqrt{d_{k}}}\right)$$

Where the scaling factor is represented as $\frac{1}{\sqrt{d_{k}}}$ and $QK^{T}$ denotes the transposition of Q and K . In scaled dot product attention, the input matrix is denoted as I and the attention mechanism is expressed based on Equations 9–13) as follows:


(9) $$I={\left[I_{1,}I_{2,}\ldots ,I_{h}\right]}_{t\times h}$$

(10) $$Q_{t\times a}=sigmoid\left(IW_{q\left(h\times a\right)}\right)$$

(11) $$K_{t\times a}=tanh\left(IW_{k\left(h\times a\right)}\right)$$

(12) $$S_{t\times t}=softmax\;(sigmoid\left(QK^{T}\right)$$

(13) $$O_{t\times h}=\sum^{}\left(I⊗S^{T}\right)$$

Where the query and the key matrices are represented as Q and K respectively. The trainable weights are represented as $W_{q}$ and $W_{k}$ , the attention score matrix is denoted by S and the output is represented as O . The length of the time step, dimension of the hidden unit, and secondary dimension of Q and K is represented as t,h and a respectively. The significant point, (i.e.) the matrix O , needs similar dimensionalities, represented as I and it achieves this by performing modification in scaled dot product attention. Initially, I is utilized as the value matrix without multiplying the weighted matrix. Then, the element-wise product is utilized to evaluate O based on Equation 13. The scaling factor $\frac{1}{\sqrt{d_{k}}}$ was neglected because the value was not large. In the last stage, the scaled-dot product attention layer was integrated with the T-LSTM which calculates the weights of the attention layer at every individual step based on the hidden state of T-LSTM and the features of the image patches. The attention layer evaluates the weight of the contextual vector which is obtained as a weighted sum of image patches. By using the context vectors, the model develops the ability to focus on the various regions of tomatoes based on disease characteristics. The integration of the attention mechanism has the ability to focus on relevant regions of the leaf image and it permits the model to selectively focus on the appropriate regions. This enhances its ability to discriminate among different classes of tomato leaf disease.

### 3.6 Experimental setup

The evaluation of the proposed T-LSTM with an attention mechanism was executed in Python 3.9 software that included the Keras 2.12.0 library for constructing the T-LSTM. The system requirements were 16 GB RAM, Intel i9 computer, and Windows 10 OS. The mathematical formulations to evaluate the performance metrics are represented in **Table 2**.

**Table 2:** Performance metrics.

Metrics	Formulae
Accuracy (A)	A=TP+TNTP+TN+FP+FN
Precision (P)	P=TPTP+FP
Recall (R)	R=TPTP+FN
F-1 score (F)	F=2×P×RP+R
Dice coefficient (D)	$D=\frac{TP\times 2}{TP\times 2+FP+FN}$
Jaccard (J)	$J=\frac{target\cap^{\;}prediction}{target\;\cup^{\;}prediction}$

Where the true positives and true negatives are characterized as TP and TN , and the false positives and false negatives are represented as FP and FN respectively. For the analysis results, the suggested T-LSTM was compared with state-of-the-art techniques such as RNN, deep belief networks (DBN), and LSTM which are clearly described in the following section.

## 4 Results

This section provides a detailed overview of the outcomes attained when evaluating the proposed T-LSTM with an attention mechanism. The results are assessed by comparing the efficacy of the proposed classifier with the other classification models listed in related works.

### 4.1 Performance analysis

Here, the performance of different segmentation, feature extraction, and classification techniques are presented. The collected tomato PlantVillage data were used to assess the efficiency of T-LSTM with an attention mechanism.

#### 4.1.1 Evaluation of different segmentation techniques

In this section, the efficiency of the segmentation technique (i.e., U-Net) is analyzed and compared with state-of-the-art methods for segmenting the images. The results were assessed by analyzing the segmentation performance of images obtained from the PlantVillage dataset. **Table 3** shows the experimental outcome attained when comparing U-Net with other state-of-the-art techniques such as FCN, SegNet, Mask-RCNN, RefineNet, and ENet.

**Table 3:** Comparison of different segmentation techniques.

Method	Segmentation accuracy (%)	Dice coefficient	Jaccard
FCN	96.10	0.89	0.52
SegNet	95.43	0.92	0.57
Mask-RCNN	96.56	0.94	0.66
RefineNet	96.94	0.95	0.72
ENet	97.05	0.96	0.75
U-Net	97.87	0.97	0.79

**Figure 8:**
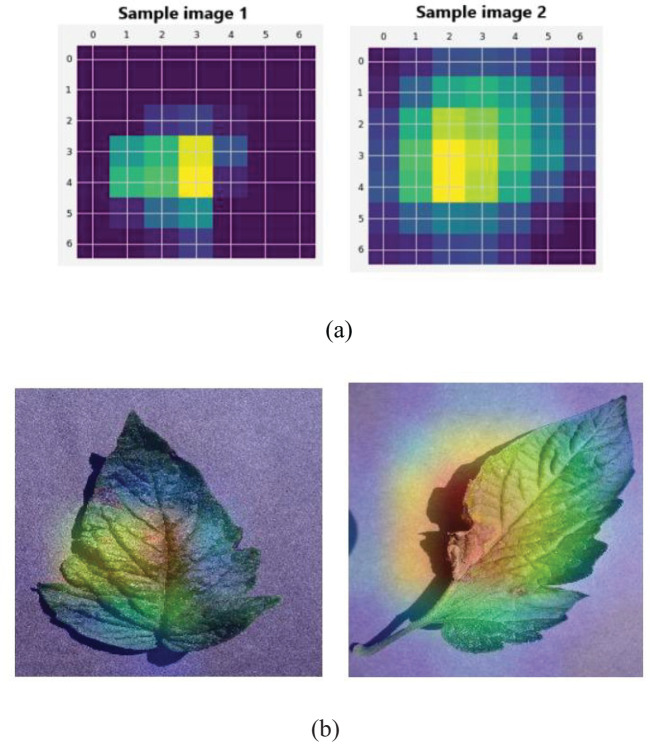
Illustration of **(A)** U-Net feature map visualization for sample images 1 and 2. **(B)** GradCAM visualization.

**Table 3** shows that the proposed segmentation method obtained better segmentation in all metrics. For example, the segmentation accuracy of the U-Net architecture utilized in this research was 97.87%, which is evidently superior to other segmentation techniques, for example, FCN (96.10%), SegNet (95.43%), Mask RCNN (96.56%), RefineNet (96.94%) and ENet (97.05%). This result is due to the U-Net architecture effectively categorizing every individual pixel of the leaf image. **Figure 8** shows an illustration of the feature maps of the UNet layers using GradCAM.

#### 4.1.2 Evaluation of different feature extraction techniques

The effectiveness of the feature extraction technique (i.e., VGG-16) was compared to other traditional models when extracting features from a segmented output. The results were evaluated by considering efficiency in the extraction of features from segmented outputs obtained from the PlantVillage dataset. **Table 4** shows the experimental outcomes attained when comparing VGG-16 with state-of-the-art techniques such as GoogleNet, AlexNet, ResNet-50, and Inception Net for the proposed classification technique. The stated metrics were used to estimate the efficiency of the VGG-16 architecture.

**Table 4:** Assessment of feature extraction methods with the proposed classifier.

Method	Accuracy (%)	Precision (%)	Recall (%)	F-1 score (%)
GoogleNet	97.01	97.24	96.34	96.79
AlexNet	97.55	96.12	96.89	96.50
ResNet-50	97.26	96.67	96.78	96.72
Inception Net	96.89	97.02	96.51	96.76
VGG-16	98.52	97.59	97.22	96.90

The results shown in **Table 4** demonstrate that the VGG-16 architecture utilized in this research outperforms other state-of-the-art techniques such as GoogleNet, AlexNet, ResNet-50, and Inception Net. The accuracy obtained by the VGG-16 architecture was 98.52% which is higher than the state-of-the-art feature extraction techniques. This result is due to VGG-16’s ability to learn hierarchical features at various levels of extraction. Moreover, the deep layers and shallow layers of VGG-16 captures high level and low-level features respectively. The usage of the 3×3 convolutional filter helps in the process of capturing refined information from the segmented output.

#### 4.1.3 Evaluation of different classification techniques with an attention mechanism

The effectiveness of the proposed classification technique (i.e., T-LSTM with an attention mechanism) utilized in this research was compared with state-of-the-art methods utilized during the stage of classification. The results were the classification of the diseased leaves and healthy leaves from the PlantVillage dataset. **Table 5** shows the experimental outcome attained when comparing the attention mechanism with state-of-the-art techniques such as RNN, DBN, LSTM, and T-LSTM. As shown in **Table 5**, the computation time per validation image for various classification models was evaluated; it shows that the proposed T-LSTM with an attention mechanism had a low computation time of 20 ms when compared with other classification models.

**Table 5:** Assessment of classification models with attention mechanism.

Method	Accuracy (%)	Precision (%)	Recall (%)	F-1 score (%)	Computation time per validation image (ms)
RNN with an attention mechanism	96.10	95.22	95.89	95.55	49
DBN with an attention mechanism	95.26	96.19	96.95	96.57	43
LSTM with an attention mechanism	97.01	96.52	96.61	96.56	35
T-LSTM with an attention mechanism	99.98	99.76	99.89	99.82	20

The outcome shown in **Table 5** shows that T-LSTM with an attention mechanism obtained superior classification outcomes in the stated measures. The performance of the classifier was evaluated based on its efficiency in classifying healthy leaves and diseased leaves. For instance, the classification accuracy of T-LSTM with an attention mechanism was 99.98%, higher than the accuracies of RNN, DBN, and LSTM with attention mechanisms of 96.10%, 95.26%, and 97.01%, respectively. The outcome of the suggested classification model was better because the scaled-dot product attention layer was integrated with the T-LSTM which calculates the weights of the attention layer at every individual step based on hidden state of T-LSTM and the features of the image patches. The attention layer evaluates the weight of the contextual vector which is obtained as the weighted sum of image patches. The incorporation of the attention mechanism gives the model the ability to focus on relevant regions of the leaf image which enhances its ability to discriminate among different classes of tomato leaf disease.

#### 4.1.4 K-fold validation for T-LSTM with an attention mechanism

The efficiency of T-LSTM with an attention mechanism for various K-values, from K=1 to K=10, was obtained. **Table 6** shows the obtained outcomes when the proposed LSTM with attention mechanism was evaluated with different K-values.

**Table 6:** Evaluation of T-LSTM with an attention mechanism for different K-values.

K-value	Accuracy (%)	Precision (%)	Recall (%)	F1 score (%)
1	94.39 ± **2.79**	95.55 ± **2.10**	96.18 ± **1.85**	96.27 ± **1.77**
2	94.12 ± **2.73**	96.89 ± **1.43**	97.27 ± **1.31**	97.08 ± **1.37**
3	96.65 ± **1.66**	97.52 ± **1.12**	96.81 ± **1.54**	96.81 ± **1.50**
4	96.89 ± **2.37**	97.37 ± **1.19**	96.94 ± **1.47**	96.62 ± **1.60**
**5**	**99.98** ± **0.01**	**99.76** ± **0.12**	**99.89** ± **0.05**	**99.82** ± **0.09**
6	97.28 ± **1.35**	96.31 ± **1.72**	97.86 ± **1.01**	97.19 ± **1.31**
7	97.11 ± **1.43**	97.28 ± **1.24**	96.93 ± **1.88**	97.49 ± **1.16**
8	97.41 ± **1.28**	96.94 ± **1.56**	96.19 ± **1.85**	96.56 ± **1.63**
9	96.57 ± **1.70**	96.64 ± **1.56**	95.51 ± **2.19**	95.83 ± **1.19**
**10**	**96.76** ± **1.61**	**97.25** ± **1.25**	**97.77** ± **1.06**	**97.77** ± **1.02**
Average	96.71 ± 1.63	97.15 ± 1.30	97.13 ± 1.38	97.14 ± 1.34

Bold*, indicates best values and its corresonding deviation.

**Figure 9:**
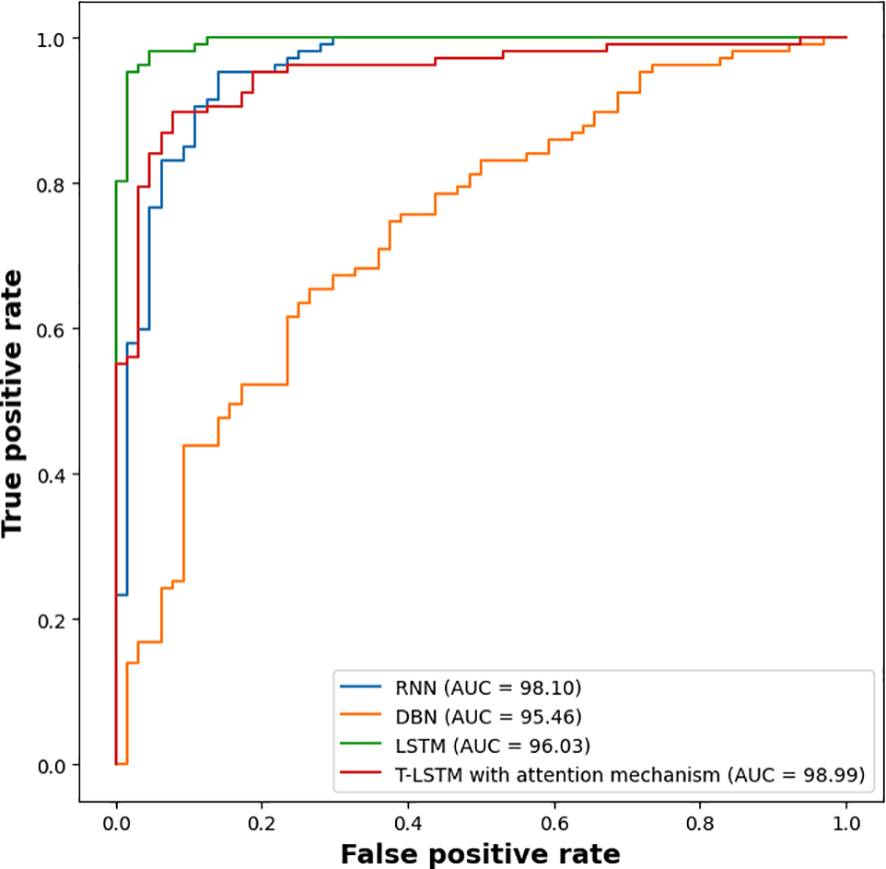
ROC graph to compute the efficiency of the classifier.

**Figure 10:**
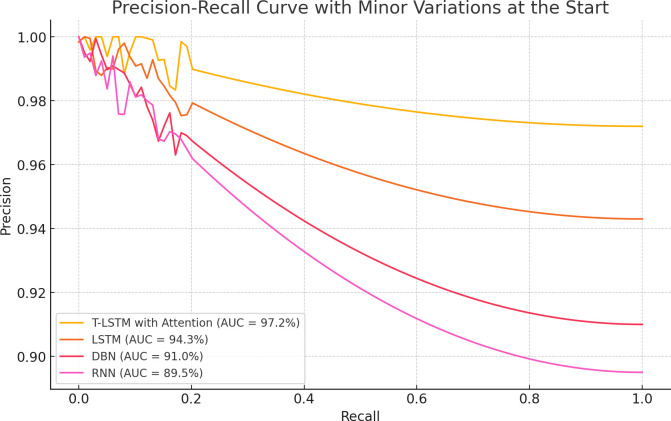
Precision-Recall Curve for K-fold validation (K=10).

The experimental outcomes shown in **Table 6** demonstrate the outcomes achieved when assessing the suggested classifier for different K-values. The proposed classifier achieved the best results when the K-value was 5, achieving an accuracy of 99.98%. The efficiency of the proposed classifier was evaluated by a receiver operational characteristics (ROC) curve, as shown in **Figure 9**. The area under the curve (AUC) value of RNN was 98.10, DBN was 95.46, LSTM was 96.03, and that of the proposed classifier was 98.99. The ROC curve presents the quality of the classifications by showing the true positive rate (TPR) and false positive rate (FPR). **Figure 10** illustrates the precision-recall curve.

#### 4.1.5 Independent analysis

**Figure 11:**
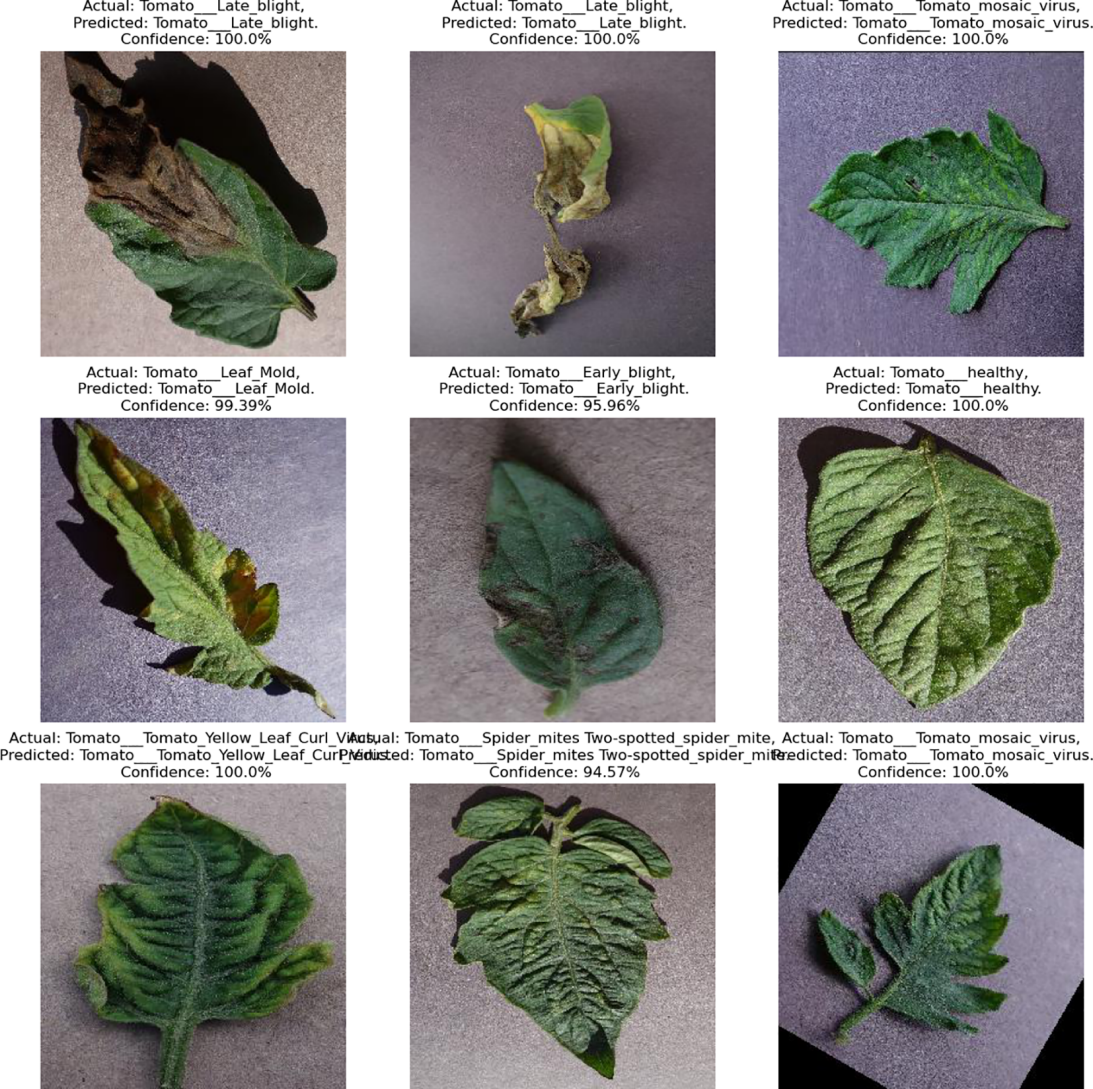
Real-time image 1 for independent analysis.

**Figure 12:**
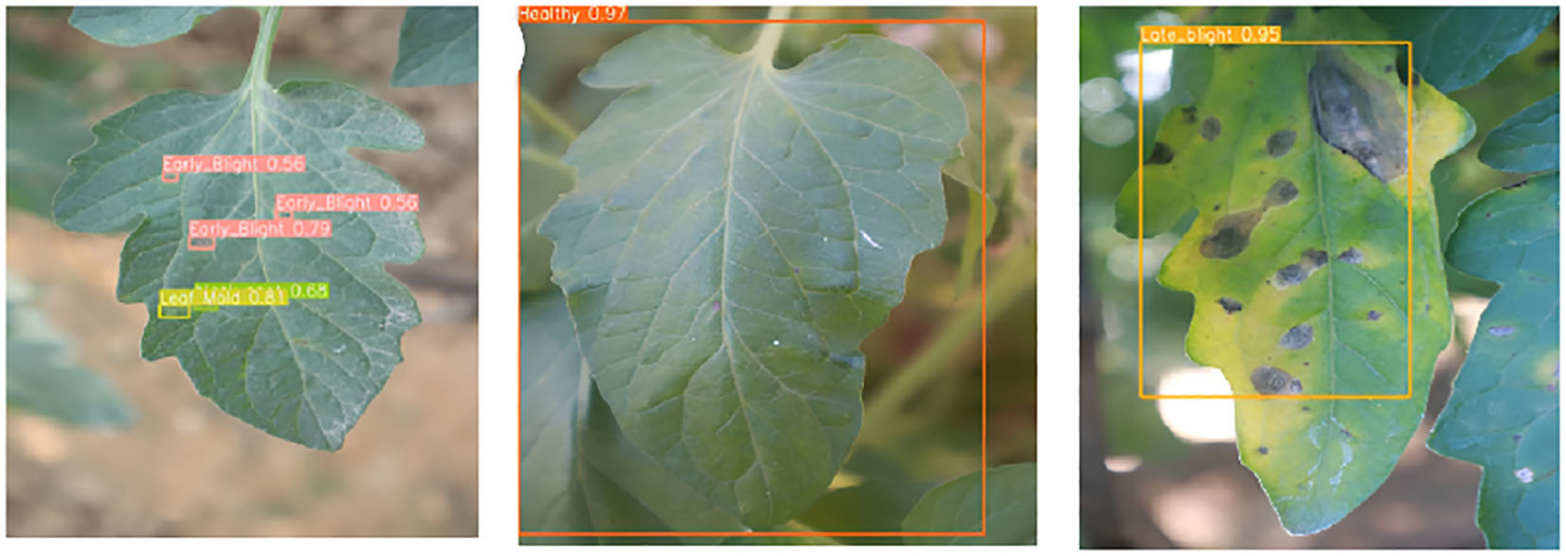
Real-time image 2 for independent analysis.

This independent analysis was validated by obtaining real-time images, with only 9 images gathered because of logistical challenges. In particular, retrieving varied agricultural backgrounds, organizing with farmers, and capturing high-quality images under changeable environments proved challenging. **Figures 11**, **12** show the collected real-time images 1 (https://www.kaggle.com/datasets/ashishmotwani/tomato/data) and 2 (https://www.kaggle.com/datasets/farukalam/tomato-leaf-diseases-detection-computer-vision) for the independent analysis. The collected images had different confidence rates, ranging from 94% to 100% for real-time image 1. During the observation of real-time image 2, there were significant results for different regions of the leaf with various categories, namely, healthy (0.97), late-blight (0.95), early-blight (0.56), and leaf mold (0.81).

### 4.2 Comparative analysis

The main objective of this research was to effectively classify tomato leaves as diseased or healthy using T-LSTM with an attention mechanism. The performance of the proposed classification framework was evaluated with existing techniques such as C-GAN DenseNet (Abbas et al., 2021), CNN with transfer learning (Ahmad et al., 2021), DCGAN-GoogleNet (Wu et al., 2020), IBSA-Net (Zhang et al., 2023), and BotanicX-AI (Bhandari et al., 2023). **Table 7** presents the outcomes when comparing the recommended classification technique with existing ones.

**Table 7:** Comparing the classification efficiency of the proposed classifier with existing ones.

Methods	Accuracy (%)	Precision (%)	Recall (%)	F1 score (%)
C-GAN DenseNet (Abbas et al., 2021)	97.11	97	97	97
CNN with transfer learning (Ahmad et al., 2021)	99.69	DNA	99.40	99.62
DCGAN-GoogleNet (Wu et al., 2020)	94.33	DNA	DNA	DNA
IBSA-Net (Zhang et al., 2023)	99.4	98.9	99.3	99.1
BotanicX-AI (Bhandari et al., 2023)	98.28	DNA	DNA	DNA
T-LSTM with an attention mechanism	99.98	99.76	99.89	99.82

^*^DNA, Data not available.

The experimental outcomes shown in **Table 6** demonstrate that the suggested classification approach had improved outcomes in the stated measures when compared with existing techniques. For instance, the accuracy of T-LSTM with an attention mechanism was 99.98%, higher than conventional C-GAN DenseNet (Abbas et al., 2021), CNN with transfer learning (Ahmad et al., 2021), DCGAN-GoogleNet (Wu et al., 2020), IBSA Net (Zhang et al., 2023), and BotanicX-AI (Bhandari et al., 2023) with classification accuracies of 97.11%,99.69%, 94.33%, 99.4%, and 98.28% respectively. This was due to the transductive learning introduced in the LSTM architecture and that scaled dot product attention layer, which was integrated in T-LSTM, that computes the weights at each step on the basis of the hidden state and the features of image patches. The attention layer computes the weight of the contextual vector that is acquired as the weighted sum of image patches. The combination of the attention layer in the architecture of T-LSTM allows the model to focus on the appropriate regions of disease-affected partitions and differentiate between the classes of tomato leaf disease.

## 5 Discussion

This research provides effective classification of tomato leaf disease using T-LSTM with an attention mechanism and this was evaluated using the tomato PlantVillage dataset. The proposed T-LSTM with an attention mechanism obtained 99.98% accuracy which highlights its potential in revolutionizing tomato leaf disease detection. Similarly, this research contributes to various domains by demonstrating the efficiency of transductive learning in describing complex disease forms. In the same way, it delivers a comprehensive assessment with state-of-the-art models, confirming the dominance of the suggested T-LSTM with an attention mechanism. When compared to existing models such as C-GAN DenseNet (Abbas et al., 2021), CNN with transfer learning (Ahmad et al., 2021), DCGAN-GoogleNet (Wu et al., 2020), and IBSA-Net (Zhang et al., 2023), the presented outcomes show an enhanced accuracy of our model by eliminating potential misclassifications. Among all the existing models, the proposed classifier accomplished a classification accuracy of 99.98%. The proposed classifier accuracy was superior to existing techniques including C-GAN DenseNet, CNN with transfer learning, DCGAN-GoogleNet, and IBSA-Net with accuracies of 97.11%, 99.69%, 94.33%, and 99.4%, respectively. Moreover, the proposed approach achieved better results in the remaining metrics, namely, precision, recall, and F-1 score. The integration of the attention layer in T-LSTM architecture focuses on a specified region of the disease-affected portions and helps in the detection of diseased and healthy leaves.

The major significance of this study is to provide an early disease detection model which allows for timely interventions, minimizing crop damage and the financial burden on the farmers. Furthermore, this research presents a revolutionary method for tomato leaf disease classification which has incomparable efficiency and accuracy. Moreover, this impact will be experienced throughout the agricultural sector from farmers to policymakers.

## 6 Conclusion

In this research, an effective classification approach known as T-LSTM with an attention mechanism was introduced to classify diseased tomato leaves and healthy tomato leaves. Data acquisition was performed using the PlantVillage dataset and pre-processing was done through image resizing, color enhancement, and data augmentation. The pre-processed data was then processed in the segmentation stage with the help of U-Net architecture. After segmentation, VGG-16 architecture was used for extraction, and then classification was made by the proposed T-LSTM with an attention mechanism. The T-LSTM was based on a transductive learning approach and the scaled dot product attention mechanism that evaluates the weights of each step based on the hidden state and image patches. The outcomes show that proposed classification technique accomplished a better classification accuracy of 99.98% when compared with existing techniques, namely, C-GAN DenseNet, CNN with transfer learning, DCGAN-GoogleNet, and IBSA-Net. In the future, the efficiency of the proposed classifier should be evaluated with real-time datasets.

## Data Availability

The original contributions presented in the study are included in the article/supplementary material. Further inquiries can be directed to the corresponding author.
